# Evolution des syndromes électrocliniques chez des jumeaux dizygotes: de l'enfance à l'adolescence, à propos d'une observation

**DOI:** 10.11604/pamj.2015.20.48.5916

**Published:** 2015-01-19

**Authors:** Marcellin Bugeme, David Mulumba Kadiebwe, Placide Kambola Kakoma, Olivier Mukuku

**Affiliations:** 1 Faculté de Médecine, Université de Lubumbashi, Lubumbashi, République Démocratique du Congo; 2 Centre Neuropsychiatrique Dr Joseph Guislain/Frères de la Charité, Lubumbashi, République Démocratique du Congo

**Keywords:** Epilepsie, syndrome électroclinique, jumeaux dizygotes, évolution, epilepsy, electroclinical syndrome, dizygotic twins, evolution

## Abstract

Les syndromes électrocliniques ont une prédisposition génétique très variable et leur évolution de l'enfance vers l'adolescence ou l’âge adulte n'est pas bien définie. Nous rapportons ici une observation mettant en jeu deux jumeaux dizygotes âgés de 19 ans ayant présenté un phénotype épileptique différent pendant l'enfance (épilepsie avec absences myocloniques et épilepsie-absence de l'enfant) mais une concordance phénotypique (épilepsie à crises généralisées tonico-cloniques prédominantes) au cours de l'adolescence. Cette observation plaide en faveur d'une implication génétique dans l’évolution de ces syndromes électrocliniques.

## Introduction

L´épilepsie est la pathologie neurologique la plus fréquente chez l´adolescent. Sa prévalence varie entre 1,5 et 2%. Il peut s´agir d´épilepsie de l´enfance qui persiste à l´adolescence ou de syndrome débutant à cet âge [1]. C'est une maladie chronique dont l´étiologie est hétérogène, et parmi les nombreuses causes de l´épilepsie, les facteurs génétiques y jouent un rôle important [2]. Les études de jumeaux épileptiques permettent d'estimer entre 40 à 80% la part de la génétique dans l’épileptogenèse [3–5]. La plupart d’épilepsies chez les jumeaux ont une hérédité complexe et sont multifactorielles. L’épilepsie résulte alors de l'action conjointe de facteurs exogènes environnementaux et de gènes (appelés gènes de susceptibilité) qui permettent l’émergence de la maladie. Cependant, même pour les épilepsies ayant une composante génétique forte (les formes monogéniques d’épilepsies en sont le modèle), les facteurs environnementaux peuvent également intervenir [6]. L'incidence de l’épilepsie chez les jumeaux en Inde était de 73,3 pour 1000 paires de jumeaux [7].

Nous rapportons ici une observation mettant en jeu deux jumeaux dizygotes âgés de 19 ans ayant présenté un phénotype épileptique différent pendant l'enfance (épilepsie avec absences myocloniques (EAM) et épilepsie-absence de l'enfant (EAE)) mais une concordance phénotypique (épilepsie à crises généralisées tonico-cloniques prédominantes) au cours de l'adolescence.

## Patient et observation

Il s´agit d´une observation clinique impliquant deux jumeaux dizygotes épileptiques de 19 ans d’âge issus d´une famille de bas niveau socioéconomique dont l’étiologie de l´épilepsie est présumée génétique (pas d'anomalie structurale et métabolique mise en évidence). Ils sont sans antécédents néonataux ni pathologiques notables. Leur croissance staturo-pondérale fut normale et les étapes du développement psychomoteur ont été acquises dans les délais normaux. Amenés dans notre centre neuropsychiatrique par leur mère, ils présentent des crises convulsives depuis plusieurs années et le début a été marqué par des crises absences pendant l'enfance. Aucun antécédent de convulsions ou d’épi¬lepsie n'est relevé dans les familles paternelle et maternelle.

**Patiente A**, sœur jumelle du patient B, présente des crises d'absences depuis l’âge de 3 ans. Leur mère signale qu’à cet âge l'enfant pouvait cesser brusquement ce qu'elle était en train de faire, et présentant un regard fixe et qui duraient quelques secondes avant que l'enfant reprenne complètement la conscience. Aucun médicament anti-épileptique ne lui avait jamais été administré jusqu’à l’âge de 11 ans où le traitement fait de carbamazépine et d'hydergine lui avait été prescrit suite à l'apparition des crises convulsives, dans un centre de santé. Après une année de traitement, il y a eu interruption brutale de ce traitement par la mère qui avait cru que la maladie de sa fille était d'ordre mystique et qu'elle ne pouvait pas guérir par les médicaments. Le complément d´anamnèse notait des crises convulsives tonico-cloniques généralisées avec révulsion oculaire, émission d´écume et perte d'urine. Chaque fois après les crises, la patiente ne se souvient pas de tout ce qui précède. Une fatigue postcritique est également notée. Les crises durent environ 10 minutes avec une fréquence journalière de plus des 10 crises.

**Figure 1:**
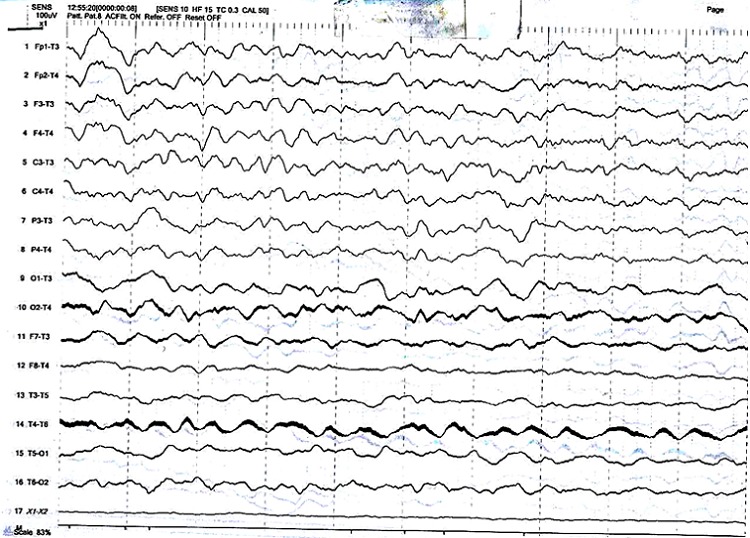
Tracé EEG de la patiente A montrant des activités lentes pathologiques généralisées dans le domaine de Delta et l'abondance en éléments lents diffuse

L'examen somatique général est sans parti¬cularité et l'examen neurologique est entière¬ment normal. L’électro-encéphalogramme (EEG) standard réalisée mettait en évidence un tracé gravement perturbé par sa lenteur (activités lentes pathologiques généralisées dans le domaine de Delta) et par l´abondance en éléments lents diffus (Figure 1). Le diagnostic d´épilepsie à crises généralisées tonico-cloniques prédominantes a été posé et l'EAE a été retenu comme syndrome électroclinique présenté pendant son enfance. Un traitement fait de Phénobarbital (100 mg par jour et le soir) a été prescrit avec une recommandation et insistance de la prise régulière de ce médicament.

**Patient B** a commencé à présenter des crises d'absence depuis l’âge de 7 ans, au cours desquelles leur mère constatait des secousses musculaires des membres supérieurs et inférieurs avec élévation de ces derniers accompagnés de perte de contact avec l'entourage et de perte d'urines. Ces crises étaient de durée et de fréquence plus élevées que celles observées chez sa sœur jumelle. Ceci motivera leur mère à consulter un centre de santé et un traitement fait de carbamazépine a été instauré. Ce traitement avait été pris pendant plus d'une année et aucune amélioration n'avait été notée, et fut arrêté sur décision de sa mère. Cinq années plus tard (à 12 ans d’âge), il y a eu apparition des crises convulsives comme cela fut chez sa jumelle et ont tous deux étaient amenés chez un tradipraticien. Leur mère refusait que ses enfants poursuivent le traitement car elle croyait que leur maladie était d´ordre mystique.

**Figure 2:**
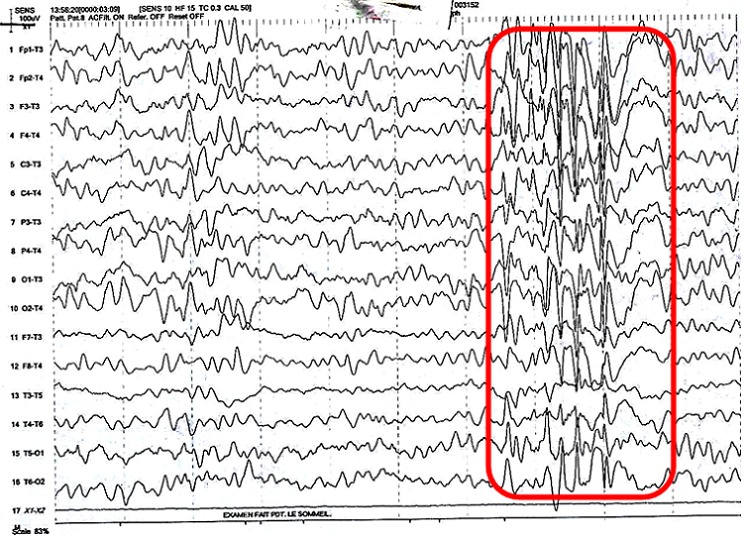
Tracé EEG du patient B montrant des activités lentes pathologiques dans les domaines de delta et thêta, généralisés et symétriques et des activités de pointes et de pointe-ondes sans prédominance de côté

Le complément d´anamnèse notait des crises convulsives qui commençaient par la déviation de la bouche suivie des mouvements tonico-cloniques généralisées de plus de 8 minutes de durée, avec révulsion oculaire et une perte d'urine. La fréquence des crises était de plusieurs crises par semaine. L´examen neurologique montrait une altération des fonctions intellectuelles, avec un retard mental léger (quotient intellectuel calculé à 65). L'EEG standard réalisée mettait en évidence un tracé compatible avec une activité épileptiforme avec existence des activités lentes pathologiques dans les domaines de delta et thêta, généralisés et symétriques et celle des activités de pointes et de pointe-ondes sans prédominance de côté (Figure 2).

Au vu de tous ces éléments, un diagnostic d´épilepsie à crises généralisées tonico-cloniques prédominantes avait été posé et le syndrome électroclinique retenu pour l'enfance est l'EAM. Le phénobarbital a été prescrit à une dose de 100 mg par jour. Après une année de suivi médical régulier, l’évolution de ces jumeaux est marquée par une amélioration clinique.

## Discussion

La génétique demeure un challenge dans les pays à ressources limitées où la réalisation d'une analyse du caryotype est inaccessible. Les syndromes électrocliniques ont une prédisposition génétique très variable et leur évolution de l'enfance à l'adolescence ou à l’âge adulte n'est pas bien définie.

Le diagnostic d´épilepsie à crises généralisées tonico-cloniques prédominantes chez nos patients a été établi selon la classification de la Ligue Internationale de Lutte contre l'Epilepsie (ILAE) de 2010 [8] et leur phénotype épileptique était concordant. Mais quand nous nous référons à l'hétéro-anamnèse, en fonction de l’âge d'apparition tel que les classe l'ILAE, les syndromes électrocliniques présentés pendant l'enfance par ces jumeaux étaient phénotypiquement discordants et correspondaient à l'EAM chez le garçon et l'EAE chez la fille. Selon la littérature, le taux de concordance phénotypique est significativement moins élevé chez les jumeaux dizygotes que chez les jumeaux monozygotes [3, 9, 10] et au cours des épilepsies absences, la concordance phénotypique est de 27% chez les jumeaux dizygotes et de 74% chez les jumeaux monozygotes [11]. L'EAM, assez rare et décrite initialement comme une forme clinique particulière de l´épilepsie-absence de l´enfant, a été ultérieurement reconnue comme entité à part compte tenu de la prépondérance des absences myocloniques comme seule ou la plus importante forme de crises, et de l´impact défavorable sur la cognition [12, 13].

Gargouri rapporte une observation de deux jumeaux monozygotes qui ont présenté une persistance d'EAE jusqu’à l’âge adulte [14]. Dans notre observation, il y a eu, non pas une persistance des épilepsies de l'enfance mais une évolution vers une épilepsie de l'adolescence. Dans les EAM, Bureau souligne que les crises peuvent complètement disparaître dans 37,5% des cas [15] et par contre, chez d'autres patients, le pronostic tend à être très défavorable, avec la persistance des absences myocloniques ou bien l´évolution vers une forme plus sévère [12, 15]. Selon Grosso, les taux de rémission de l'EAE rapportés dans la littérature sont varient de 33 à 79% et dépendent du type de critères diagnostic adoptés et de la diversité des durées de suivi [16].

Bien que nos patients aient été à traités à tort dans le passé par la carbamazépine reconnue comme molécule aggravant les épilepsies absences [17–19], il nous semble difficile de confirmer la véritable cause de leur mauvaise évolution. Est-ce une implication de facteurs génétiques dans l’évolution de ces épilepsies de l'enfance à l'adolescence? Nous avons porté le choix sur le phénobarbital vu son efficacité dans les crises convulsives généralisées, son faible coût et sa prescription en monoprise quotidienne [20], ce qui permet une bonne observance thérapeutique chez nos patients.

## Conclusion

En passant de l'enfance à l'adolescence ou à l’âge adulte, l’évolution des épilepsies absences vers une forme plus sévère peut être sous l'influence de facteurs génétiques mais aussi la prescription à tort de la carbamazépine. Cette observation de jumeaux dizygotes avec EAM et EAE ayant évolué vers une épilepsie à crises généralisées tonico-cloniques prédominantes à l'adolescence plaide en faveur d'une implication génétique dans l’évolution de ces syndrome électrocliniques.
